# Prospective Intraindividual Comparison of Ga-68 PSMA and F-18 FDG Dual-Tracer PET/CT Imaging in Evaluation of High-Grade Prostate Cancer

**DOI:** 10.1055/s-0046-1825755

**Published:** 2026-07-15

**Authors:** Harini Koorma, Mohan Roop Jayanthi, Suneetha Batchu, Anuradha Sekaran, Rajesh Kumar Reddy, Mallikarjuna Chiruvella

**Affiliations:** 1 Department of Nuclear Medicine and PET-CT, Asian Institute of Gastroenterology (AIG) Hospitals, Hyderabad, Telangana, India; 2 Department of Pathology, Asian Institute of Gastroenterology (AIG) Hospitals, Hyderabad, Telangana, India; 3 Department of Urology, Asian Institute of Nephrology and Urology (AINU), Hyderabad, Telangana, India

**Keywords:** prostate-specific membrane antigen (PSMA), fluorodeoxyglucose (FDG), International Society of Urological Pathology (ISUP), prostate cancer, Gleason score

## Abstract

**Introduction:**

High-grade prostate cancer (International Society of Urological Pathology [ISUP] grade groups 4 and 5) is often aggressive and associated with early metastatic spread. Gallium-68 (Ga-68) prostate-specific membrane antigen (PSMA) positron emission tomography/computed tomography (PET/CT) is widely adopted for prostate cancer imaging due to its high sensitivity, while F-18 fluorodeoxyglucose (FDG) PET/CT may provide complementary metabolic information. This study investigates and compares the diagnostic performance of these two modalities and evaluates the incremental value of dual-tracer PET/CT imaging.

**Methods:**

This prospective observational study included 51 patients with biopsy-proven, high-grade prostate adenocarcinoma (ISUP grade groups 4 and 5) undergoing both Ga-68 PSMA and F-18 FDG PET/CT within 1 week. Detection rates for primary tumors, nodal, and distant metastases were analyzed. Maximum standardized uptake values (SUVmax), lesion-to-aorta and lesion-to-muscle ratios, and serum prostate-specific antigen (PSA) levels were correlated.

**Results:**

Ga-68 PSMA PET/CT demonstrated superior detection rate for primary tumor detection (90.2%) compared to F-18 FDG PET/CT (66.7%, *p* = 0.004). For pelvic lymph nodes, detection rates were numerically higher with PSMA PET/CT (76.2% vs. 59.5% for FDG PET/CT), although this difference did not reach statistical significance; for extrapelvic lymph nodes, 95.2% (PSMA) versus 52.4% (FDG, *p* = 0.02); and for bone lesions, 100% (PSMA) versus 77.8% (FDG, *p* = 0.009). In ISUP grade 4, primary detection was 94.4% (PSMA) versus 50% (FDG, *p* = 0.003); in grade 5, 87.9% (PSMA) versus 75.8% (FDG). Dual-tracer imaging improved overall detection of primary tumors and pelvic lymph nodes to 100 and 90.4%, respectively. A mild correlation was observed between PSA levels and PSMA SUVmax ( *r* = 0.297, *p* = 0.034). Strong positive correlations were found between SUVmax and both lesion-to-aorta and lesion-to-muscle ratios for both tracers. Interreader agreement was excellent for PSMA ( *κ* = 1.00) and high for FDG ( *κ* = 0.95).

**Conclusion:**

Ga-68 PSMA PET/CT demonstrated high detection rates for detecting primary and metastatic lesions in high-grade prostate cancer. F-18 FDG PET/CT offers complementary metabolic information, particularly valuable in ISUP grade 5 and dedifferentiated tumors. Dual-tracer PET/CT enhances diagnostic accuracy and may facilitate personalized treatment strategies in aggressive prostate cancer.

## Introduction

Prostate cancer is primarily a disease of elderly men above 65 years of age. Prostate cancer was the second most commonly diagnosed cancer and the fifth leading cause of cancer death among men worldwide in 2020. 1 By 2040, the global burden of prostate cancer is projected to rise substantially, with nearly 2.9 million new cases annually and a marked increase in prostate cancer-related mortality. 2

Prostate cancer is a biologically and clinically heterogeneous disease that makes imaging evaluation a challenging task. With the advent of positron emission tomography (PET), there has been use of several radiotracers such as F-18 fluorodeoxyglucose (FDG) PET, gallium-68 (Ga-68) prostate-specific membrane antigen (PSMA), C-11 choline, and F-18 choline for prostate cancer imaging. However, choline-based PET/computed tomography (CT) reported that there are low sensitivity and specificity especially in cases of low serum prostate-specific antigen (PSA) levels. PSMA is a cell surface protein with high expression in prostate cancer cells. 3 4 Preliminary studies have hypothesized that Ga-68 PSMA PET/CT detection rates will increase with rising PSA levels, increased pathological Gleason score, and tumor size. 5

F-18 FDG remains the workhorse in PET imaging, however, with very limited applications for prostate cancer. Most prostate cancers use fatty acid metabolism or fructose rather than glucose resulting in low glucose metabolism. Localization of primary prostate cancer using F-18 FDG PET/CT usually demonstrates low FDG uptake, sometimes, incidental high FDG uptake is also seen. A systemic review and meta-analysis have showed a pooled prevalence of 1.8% for incidental high prostate uptake of FDG, having a significant malignancy risk of 62% in biopsy-proven cases. 6

F-18 FDG PET/CT has low overall sensitivity and specificity in the detection of primary disease. However, a higher sensitivity of FDG PET/CT in the detection of primary disease has been reported in patients with increasing Gleason score, clinical stage, and serum PSA level, suggesting that FDG PET/CT might be useful in selected cases. The purpose of this study was to compare the diagnostic accuracy of Ga-68 PSMA and F-18 FDG PET/CT in the detection of primary, nodal, and distant metastases in high-grade prostate cancer and also to look for any statistical significance on addition of F-18 FDG along with Ga-68 PSMA PET/CT in any particular subset of patients with high-grade prostate cancer.

## Materials and Methods

This is a prospective observational study where 51 patients had undergone Ga-68 PSMA and F-18 FDG PET/CT imaging from May 2023 to December 2024 in our institution. The inclusion criteria are biopsy-proven recently diagnosed cases of high-grade carcinoma prostate. Patients with following characteristics where excluded: patients refusing to give written informed consent; uncontrolled diabetics, as F-18 FDG uptake may be hampered in such patients; and patients who had received any prior treatment for prostate cancer. This study was approved by the Ethics Committee of our hospital.

### Ga-68 PSMA and F-18 FDG PET/CT Imaging

Patients were on fasting for at least 6 hours. No antidiabetic medication on the day of study with recent serum creatinine as standard protocol. Note that 296 to 370 MBq (8–10 mCi) of F-18 FDG is injected intravenously provided the blood sugar levels are less than 200 mg/dL. Thereafter, patients rest in an isolated quiet room for 45 to 60 minutes. 68Ga-PSMA-11 synthesis was performed as previously described. 7 Note that 126 to 154 MBq (3–4 mCi) of Ga-68 PSMA is given as an intravenous bolus. Patients should be well-hydrated before the study. Voiding immediately before imaging acquisition is recommended. Contrast CT scan was obtained as a part of PET/CT protocol on multislice (16 slice) CT with 1.5 mm slice thickness with intravenous nonionic contrast injection and was followed by PET imaging. Acquisition time was 45 sec/bed position. Delayed PET/CT of pelvis, 3 hours postinjection, was also taken wherever needed.

### Interpretation of Ga-68 PSMA PET/CT Imaging

Two board-certified physicians specializing in nuclear medicine independently evaluated the imaging results derived from Ga-68 PSMA and F-18 FDG PET/CT modalities. Standard criteria for interpreting scan positivity were applied to both scans: (1) for Ga-68 PSMA PET/CT, positivity was determined by uptake intensity greater than the normal physiological uptake in areas like the salivary glands, liver, and blood pool/background, and (2) for F-18 FDG PET/CT, positivity was defined as uptake higher than the expected physiological levels in the relevant tissue or organ compared to the background. Maximum standardized uptake values (SUVmax) of all lesions in the prostate gland were calculated, by drawing region of interest and were normalized to body weight. Tumor sizes and SUVmax for lymph nodes and organs with extranodal disease was measured for each scan.

Histopathological confirmation was available for the primary prostate lesion through biopsy. Nodal and distant metastatic lesions were interpreted based on composite imaging findings, contrast-enhanced CT correlation, tracer uptake characteristics, and consensus interpretation by the experienced nuclear medicine physicians. Therefore, the reported results represent detection rates rather than the true sensitivity or specificity values.

### Statistical Analysis

The data was collected using a study pro forma. All the data was entered into MS Excel. Data was analyzed using Statistical Package for Social Sciences (SPSS). Categorical data was presented as frequencies and percentages. Continuous data was presented as mean with standard deviation (SD). Chi-square test was used as test of significance for categorical data. Cohen's kappa coefficient ( *κ* ) was used to measure interrater agreement for qualitative data (Ga-68 PSMA PET/CT, F-18 FDG PET/CT findings). A *p* -value of less than 0.05 was considered as statistically significant.

## Results

In total, 51 patients had undergone Ga-68 PSMA and F-18 FDG PET/CT imaging from May 2023 to December 2024. The baseline characteristics of patients are summarized in Table 1 . Most patients had serum PSA levels > 20 ng/mL and belonged to International Society of Urological Pathology (ISUP) grade group 5.

**Table 1:** Baseline clinicopathological characteristics of the study population

Characteristic	Value
Age (y)	
Mean ± SD	65.4 ± 10.1
Median (IQR)	66 (60–73)
PSA level (ng/mL)	
Mean ± SD	135.9 ± 282.5
Median (IQR)	42.0 (21.8–120.0)
Serum PSA distribution, *n* = 51	
< 10 ng/mL	2
10–20 ng/mL	8
> 20 ng/mL	41
Gleason score, *n* = 51	
8	18
9	23
10	10
ISUP grade group, *n* = 51	
Grade group 4	18
Grade group 5	33

Abbreviations: IQR, interquartile range; ISUP, International Society of Urological Pathology; PSA, prostate-specific antigen; SD, standard deviation.

### Overall Detection Rates of F-18 FDG AND Ga-68 PSMA PET/CT

**Fig. 1:**
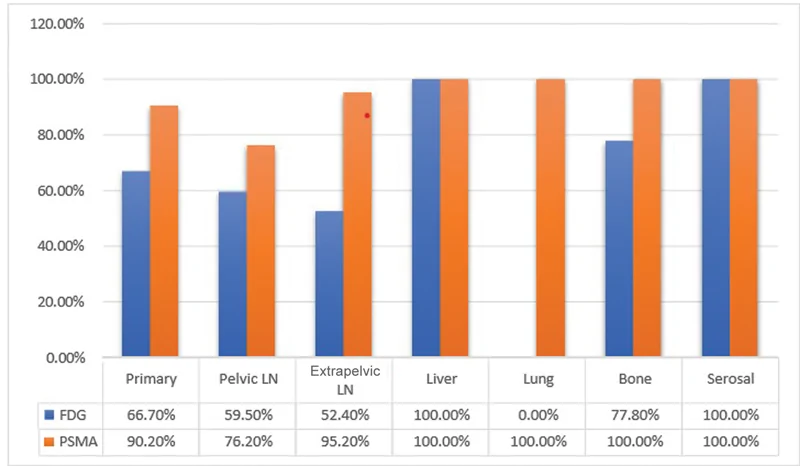
Overall detection rate of F-18 fluorodeoxyglucose (FDG) and gallium-68 (Ga-68) prostate-specific membrane antigen (PSMA) positron emission tomography/computed tomography (PET/CT).

In patients recently diagnosed with biopsy-proven, high-grade prostate adenocarcinoma (ISUP grade groups 4 and 5) and varying serum PSA levels, the detection rate for primary was significantly lower with F-18 FDG PET/CT (66.7%, 34/51) compared to Ga-68 PSMA PET/CT (90.2%, 46/51). This difference was found to be statistically significant, with a *p* -value of 0.004. F-18 FDG PET/CT showed lower detection rates than Ga-68 PSMA PET/CT for pelvic lymph node region (59.5%, 25/42 vs. 76.2%, 32/42; *p* = 0.102); extrapelvic lymph node region (52.4%, 11/21 vs. 95.2%, 20/21; *p* = 0.02); bone (77.8%, 21/27 vs. 100%, 27/27; *p* = 0.009); and lung (0%, 0/2 vs. 100%, 2/2; *p* = 0.317), as shown in Fig. 1 .

Comparisons involving lung liver and serosal/peritoneal metastases should be interpreted cautiously because of the very limited number of patients in these subgroups; therefore, these findings are descriptive in nature.

Ga-68 PSMA PET/CT detected 129 lesions in 46 of the 51 patients, and F-18 FDG PET/CT detected 95 lesions in 34 of the 51 patients. Overall, 144 lesions were detected by Ga-68 PSMA PET/CT, F-18 FDG PET/CT, or both.

### Detection Rates of F-18 FDG and Ga-68 PSMA PET/CT in ISUP Grade Group 4

**Fig. 2:**
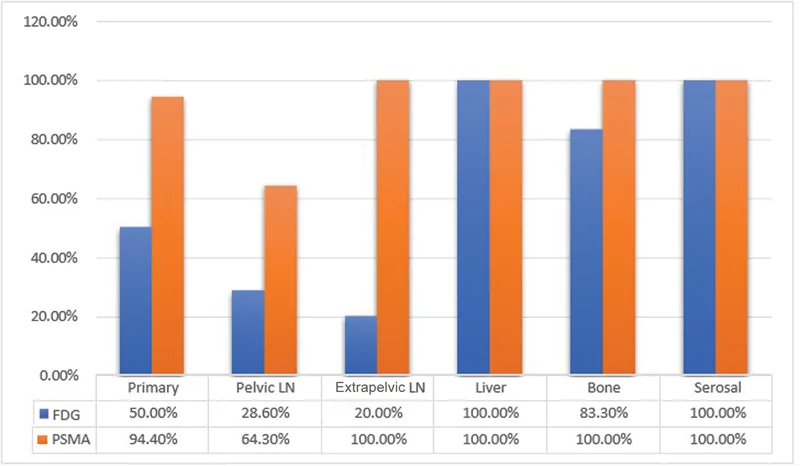
Detection rates of F-18 fluorodeoxyglucose (FDG) and gallium-68 (68-Ga) prostate-specific membrane antigen (PSMA) positron emission tomography/computed tomography (PET/CT) in grade group 4.

In patients recently diagnosed with biopsy-proven, high-grade prostate adenocarcinoma in ISUP grade group 4, the detection rate for primary was significantly lower with F-18 FDG PET/CT (50%, 9/18) compared to Ga-68 PSMA PET/CT (94.4%, 17/18). This difference was found to be statistically significant, with a *p* -value of 0.003. F-18 FDG PET/CT showed lower detection rates than Ga-68 PSMA PET/CT for pelvic lymph node region (28.6%, 4/14 vs. 64.3%, 9/14; *p* = 0.058); extrapelvic lymph node region (20%, 1/5 vs. 100%, 5/5; *p* = 0.01); bone (83.3%, 5/6 vs. 100%, 6/6; *p* = 0.29); and liver and serosal/peritoneal/omental (100%, 1/1 vs. 100%, 1/1; *p* -value not calculated because of small sample size), as shown in Fig. 2 .

### Detection Rates of F-18 FDG and Ga-68 PSMA PET/CT in Grade Group 5

**Fig. 3:**
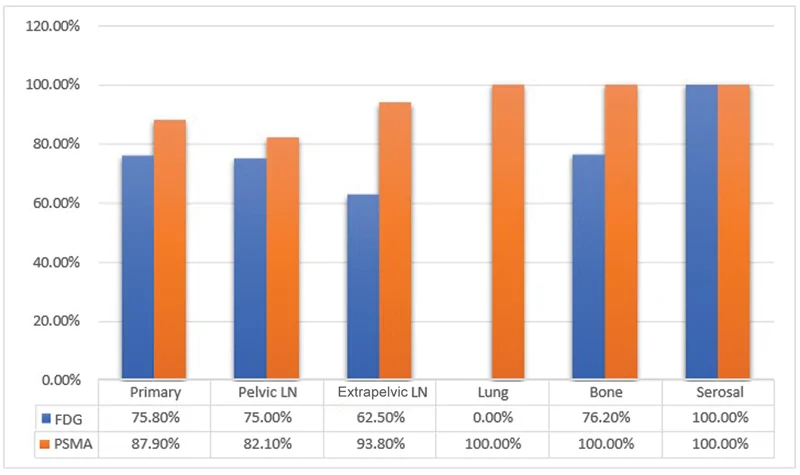
Detection rates of F-18 fluorodeoxyglucose (FDG) and gallium-68 (68-Ga) prostate-specific membrane antigen (PSMA) positron emission tomography/computed tomography (PET/CT) in grade group 5.

In patients recently diagnosed with biopsy-proven, high-grade prostate adenocarcinoma in ISUP grade group 5, the detection rate for primary was significantly lower with F-18 FDG PET/CT (75.8%, 25/33) compared to Ga-68 PSMA PET/CT (87.9%, 29/33). This difference was found to be statistically significant, with a *p* -value of 0.202. F-18 FDG PET/CT showed lower detection rates than Ga-68 PSMA PET/CT for pelvic lymph node region (75%, 21/28 vs. 82%, 23/28; *p* = 0.515); extrapelvic lymph node region (62.5%, 10/16 vs. 93.8%, 15/16; *p* = 0.033); bone (76.2%, 16/21 vs. 100%, 21/21; *p* = 0.017); lung (0%, 0/2 vs. 100%, 2/2; *p* = 0.046); and serosal/peritoneal/omental (100%, 1/1 vs. 100%, 1/1; *p* -value not calculated because of small sample size), as shown in Fig. 3 .

#### The Incremental Value of Incorporating F-18 FDG PET/CT with Ga-68 PSMA PET/CT in Staging

In the initial staging of patients, the inclusion of F-18 FDG PET/CT notably improves the detection rates of primary tumors and pelvic lymph node metastases, when compared to Ga-68 PSMA PET/CT alone. Specifically, the overall detection rates for primary lesions and pelvic lymph node metastases were increased from 90.2 to 100% and 76.2 to 90.4%, respectively, as shown in Table 2 . For patients classified within grade group 4, the addition of F-18 FDG PET/CT enhanced the detection of primary and pelvic lymph node metastases, in comparison to Ga-68 PSMA PET/CT alone. In patients within grade group 5, the addition of F-18 FDG PET/CT further improved detection of primary and pelvic lymph node metastases, when compared with Ga-68 PSMA PET/CT alone. Notably, no substantial additional benefit was observed for the detection of extrapelvic lymph node or bone metastases, as these were all successfully identified using Ga-68 PSMA PET/CT alone. Table 2 illustrates the added value of incorporation of F-18 FDG PET/CT along with Ga-68 PSMA PET/CT.

**Table 2:** Detection rates of primary tumor and pelvic lymph node metastases on 68Ga-PSMA PET/CT and 18F-FDG PET/CT according to ISUP grade group

Group	Patients ( *n* )	Site	68Ga-PSMA PET/CT, *n* (%)	18F-FDG PET/CT, *n* (%)	Both modality positive, *n* (%)
Overall	51	Primary tumor	46 (90.2)	34 (66.7)	51 (100)
		Pelvic lymph nodes	32 (76.2)	25 (59.5)	38 (90.4)
Grade group 4	18	Primary tumor	17 (94.4)	9 (50.0)	18 (100)
		Pelvic lymph nodes	9 (64.3)	4 (28.6)	11 (78.5)
Grade group 5	33	Primary tumor	29 (87.9)	25 (75.8)	33 (100)
		Pelvic lymph nodes	23 (82.0)	21 (75.0)	27 (96.2)

Abbreviations: 68Ga, gallium-68; FDG, fluorodeoxyglucose; ISUP, International Society of Urological Pathology; PET/CT, positron emission tomography/computed tomography; PSMA, prostate-specific membrane antigen.

### Correlation Between Serum PSA Levels and Primary PSMA SUVmax and FDG SUVmax in Newly Diagnosed Prostate Adenocarcinoma

A mildly positive correlation was observed between serum PSA levels and primary PSMA SUVmax in patients with newly diagnosed prostate adenocarcinoma (Spearman's correlation coefficient, 0.297; *p* = 0.034). In contrast, no significant correlation was found between serum PSA levels and primary FDG SUVmax (Spearman's correlation coefficient, 0.122; *p* = 0.393).

### Correlation Between Lesion-To-Aorta Ratio, Lesion-To-Muscle Ratio, and Primary PSMA SUVmax and FDG SUVmax in Prostate Adenocarcinoma

Both the lesion-to-aorta ratio and lesion-to-muscle ratio demonstrated a significantly positive correlation with PSMA SUVmax (Spearman's correlation coefficient, 0.898; *p* < 0.0001 and Spearman's correlation coefficient, 0.812; *p* < 0.0001, respectively). Similarly, both ratios showed a significantly positive correlation with FDG SUVmax (Spearman's correlation coefficient, 0.929; *p* < 0.0001 and Spearman's correlation coefficient, 0.826; *p* < 0.0001, respectively).

### Interreader Agreement

F-18 FDG PET/CT demonstrated marginally lower interreader agreement than Ga-68 PSMA PET/CT, with *κ* values of 0.95 and 1.00, respectively, at both the patient level and for regional assessments (both *p* = 0.001).

The two nuclear medicine physicians interpreted Ga-68 PSMA PET/CT and F-18 FDG PET/CT scans independently. Readers were blinded to the findings of the alternate tracer imaging modality during interpretation. However, basic clinical information including biopsy-proven diagnosis and serum PSA values were available at the time of reporting, reflecting routine clinical practice.

### Semiquantitative Analysis of Concordantly Positive Lesions

**Fig. 4:**
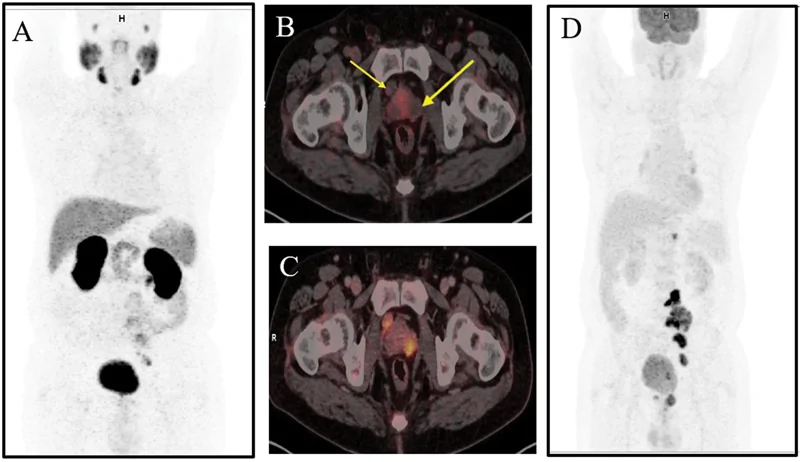
Gallium-68 (Ga-68) prostate-specific membrane antigen (PSMA) positron emission tomography/computed tomography (PET/CT). ( **A** ) Maximum intensity projection (MIP) and ( **B** ) fused PET/CT shows nontracer avid hypoenhancing lesion in the peripheral zone of the left lobe of prostate (marked by bold, larger yellow arrow) and nontracer avid perilesional lymph node (marked by thin, short yellow arrow) and few nonregional lymph nodes showing faint uptake. F-18 fluorodeoxyglucose (FDG) PET/CT ( **C** , fused PET/CT; **D** , MIP) shows intense tracer uptake in prostate lesion and also in regional and nonregional lymph nodes.

**Fig. 5:**
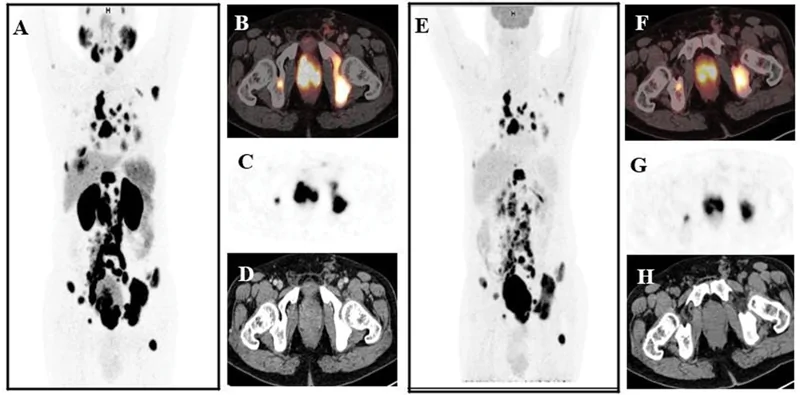
Concordance between gallium-68 (Ga-68) prostate-specific membrane antigen (PSMA) positron emission tomography/computed tomography (PET/CT) and F-18 fluorodeoxyglucose (FDG) PET/CT. ( **A** – **D** ) ( **A** , maximum intensity projection [MIP]; **B** , fused PET/CT; **C** , axial PET; **D** , axial CT) Ga-68 PSMA PET/CT showed intense increased PSMA tracer uptake in soft tissue density lesion replacing the prostate gland, regional and nonregional lymph nodes, and in skeletal metastases. ( **E** – **H)** ( **E** , MIP; **F** , fused PET/CT; **G** , axial PET; **H** , axial CT) F-18 FDG PET/CT showed intense FDG avid primary in prostate gland, regional and nonregional lymph nodes, and in skeletal metastases.

**Fig. 6:**
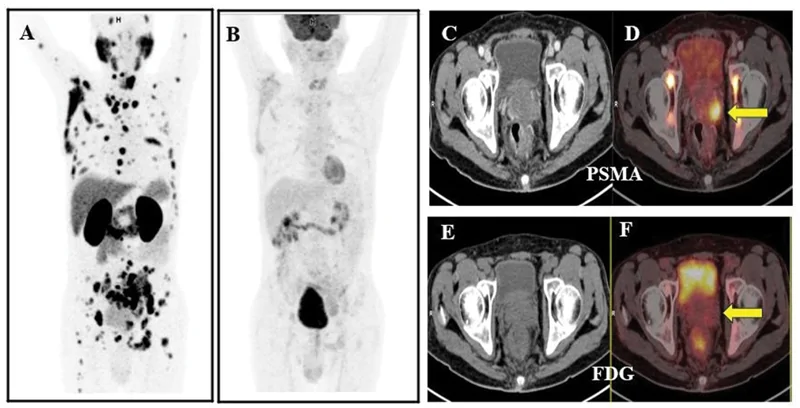
Discordance between gallium-68 (Ga-68) prostate-specific membrane antigen (PSMA) positron emission tomography/computed tomography (PET/CT) and F-18 fluorodeoxyglucose (FDG) PET/CT. ( **A** , **C** , **D** ) ( **A** , maximum intensity projection [MIP]; **C** , axial CT; **D** , fused PET/CT) Ga-68 PSMA PET/CT scan showed intense tracer uptake in the posterior peripheral basal zone of the left lobe of prostate gland and also in regional lymph nodes and in skeletal metastases. ( **B** , **E** , **F** ) ( **B** , MIP; **E** , axial CT; **F** , fused PET/CT) F-18 FDG PET/CT scan showed no significant metabolically active disease in whole body.

Among the 51 patients with a Gleason score of 8, 9, and 10, 29 patients showed positive detection both in PSMA and FDG PET/CT. Of these 29 patients, 29 had concordant positive lesions in the prostate bed, 21 had concordant positive lesions in the pelvic lymph node region, 12 had concordant positive lesions in the extrapelvic lymph node region, 27 had concordant positive lesions in the bone, and none had concordant positive lesions in other organs. The semiquantitative analysis (SUVmax) of lesion uptake was evaluated in the concordantly positive lesions as represented in Table 3 . Figs. 4 to 6 illustrate the presence of concordant and discordant lesions, respectively, as observed through Ga-68 PSMA and F-18 FDG PET/CT imaging modalities.

**Table 3:** SUVmax comparison of concordantly positive lesions on 68Ga-PSMA PET/CT and 18F-FDG PET/CT according to disease site

Site	68Ga-PSMA SUVmax, median (IQR)	18F-FDG SUVmax, median (IQR)	*p* -Value
Primary tumor	16.0 (9.3–44.25)	9.1 (6.95–16.75)	0.003
Pelvic lymph nodes	14.55 (4.85–29.15)	9.65 (5.43–12.18)	0.008
Extrapelvic lymph nodes	16.30 (11.08–35.50)	7.50 (3.32–12.08)	0.001
Bone lesions	24.75 (16.03–43.18)	9.90 (7.50–16.60)	0.18

Abbreviations: 68Ga, gallium-68; FDG, fluorodeoxyglucose; IQR, interquartile range; PET/CT, positron emission tomography/computed tomography; PSMA, prostate-specific membrane antigen; SUVmax, maximum standardized uptake value.

## Discussion

Prostate cancer, particularly high-grade prostate cancer, poses considerable challenges in clinical management due to its aggressive behavior and tendency for early metastatic spread. Accurate staging is pivotal for guiding treatment decisions and improving prognostication. Although conventional imaging modalities, such as multiparametric magnetic resonance imaging and CT, have been widely used in prostate cancer staging, molecular imaging techniques such as Ga-68 PSMA PET/CT and F-18 FDG PET/CT have emerged as promising tools for more precise detection of both primary and metastatic disease. 8 These imaging modalities offer complementary advantages, with Ga-68 PSMA PET/CT demonstrating high sensitivity for prostate cancer-specific targets, while F-18 FDG PET/CT can provide additional information regarding metabolic activity in tumors with high glycolytic activity. This prospective study sought to evaluate and compare the detection capabilities of Ga-68 PSMA PET/CT and F-18 FDG PET/CT imaging in patients with recently diagnosed high-grade prostate cancer, focusing on the identification of primary tumors, nodal involvement, and distant metastases. This study provides valuable insights into the utility of dual-tracer PET imaging for comprehensive staging.

A cohort of 51 patients, all with biopsy-proven, high-grade prostate adenocarcinoma, was analyzed. The mean age of the participants was 65.4 ± 10.1 years, with a median age of 66 years, and a mean serum PSA level of 135.9 ng/mL (SD = 282.57). Notably, a majority of patients (96%) presented with PSA levels above 10 ng/mL, aligning with previous studies that correlate elevated PSA levels with higher Gleason scores and more aggressive disease. 9 10 F-18 FDG PET/CT demonstrated marginally lower interreader agreement than Ga-68 PSMA PET/CT, with *κ* values of 0.95 and 1.00, respectively.

Our study found that the detection rate for primary tumors was significantly higher for Ga-68 PSMA PET/CT (90.2%, 46/51) compared to F-18 FDG PET/CT (66.7%, 34/51). This result is consistent with the findings of Zhou et al, 8 11 who demonstrated superior performance of PSMA-based PET imaging in detecting metastases compared to F-18 FDG PET/CT. Specifically, PSMA PET/CT showed better detection rates for pelvic and extrapelvic lymph nodes, bone, and lung metastases, identifying 129 lesions in 46 patients, while F-18 FDG PET/CT detected 95 lesions in 34 patients. The superior performance of Ga-68 PSMA PET/CT can be attributed to its specificity for prostate cancer cells, which express PSMA, enabling better localization of both primary tumors and metastases. In contrast, F-18 FDG PET/CT showed a lower detection rate overall but provided valuable additional information, particularly in patients with more advanced disease. Few studies reported a detection rate of 66 to 80% for Gleason score ≥ 7 prostate carcinoma, suggesting that glucose metabolism increases in patients with more aggressive disease. These findings are consistent with our study, which showed a detection rate of 66.7%. 12 13 In ISUP grade group 4, Ga-68 PSMA PET/CT demonstrated a significantly higher detection rate for primary tumors (94.4%) compared to F-18 FDG PET/CT (50%). The enhanced performance of PSMA PET/CT in detecting primary tumors and metastases in this cohort underscores its utility in high-risk prostate cancer staging, as also noted in previous study, 8 where they observed a higher sensitivity of PSMA PET/CT in identifying clinically significant prostate cancer compared to other imaging modalities.

Interestingly, our study showed that F-18 FDG PET/CT detection rate for primary tumors and pelvic lymph node metastases increased significantly in ISUP grade group 5 patients compared to grade group 4. This is in agreement with studies indicating that advanced prostate cancers, particularly those with high Gleason scores (ISUP grade group 5) and low serum PSA levels (often exhibit reduced PSMA PET/CT tracer uptake, suggesting a nonsecretory variant of prostate cancer) often demonstrate enhanced FDG uptake. The increase in FDG uptake is likely due to the higher expression of glucose transporter 1 (GLUT1) in aggressive prostate cancers. 14 Thus, F-18 FDG PET/CT may be especially useful in identifying metabolic changes in more aggressive, dedifferentiated prostate cancers, providing important prognostic insights. Furthermore, although F-18 FDG PET/CT is generally considered less effective for staging prostate cancer due to the low inherent FDG uptake in prostate cancer cells, 15 16 the current study highlights its incremental value in patients with high-grade disease (ISUP grade group 5). In fact, the detection of pelvic lymph node metastasis was significantly higher in ISUP grade group 5 patients, suggesting that F-18 FDG PET/CT may be more sensitive to certain biological features, such as hypoxia, which are common in high-grade tumors. 17 The hypoxic microenvironment in prostate cancer has been associated with increased tumor invasiveness, metastasis, and resistance to conventional therapies. 17 18 This biological characteristic can elevate tumor glucose metabolism, leading to increased FDG uptake and enhancing the sensitivity of F-18 FDG PET/CT in detecting metastatic lesions, especially in high-grade prostate cancer.

Among the five PSMA-negative patients in our cohort, the majority belonged to ISUP grade group 5 and demonstrated relatively increased FDG uptake, suggesting aggressive and potentially dedifferentiated tumor biology. Some of these patients also demonstrated advanced metastatic disease burden despite absent or mild PSMA expression, thereby supporting the complementary role of F-18 FDG PET/CT in identifying biologically aggressive PSMA-low/negative disease. The presence of discordant PSMA and FDG uptake patterns may reflect underlying biological heterogeneity and varying degrees of tumor differentiation, highlighting the complementary roles of these imaging modalities in aggressive prostate cancer. 19 20

The increased sensitivity of F-18 FDG PET/CT in detecting advanced prostate cancer can also be explained by its ability to highlight areas of tumor dedifferentiation, often associated with worse clinical outcomes. The study by Lavallée et al 13 supports the association between increased FDG uptake and biologically aggressive high-grade prostate cancer. In our cohort, although a mild positive correlation was observed between serum PSA levels and primary PSMA SUVmax, no significant correlation was found with FDG SUVmax, suggesting that PSA alone may not fully reflect the biological heterogeneity of aggressive prostate cancer. This suggests that while PSA remains an important biomarker, its correlation with prostate cancer aggressiveness, particularly in high-grade cases, may be influenced by various molecular and histopathological factors not captured by PSA alone. 9 10 Both the lesion-to-aorta ratio and lesion-to-muscle ratio demonstrated a significantly positive correlation with PSMA SUVmax and FDG SUVmax.

We acknowledge that PSA levels, ISUP grade, tumor burden, and treatment-naive status are biologically interrelated variables that may collectively influence tracer uptake and lesion detectability. However, robust multivariate regression analysis could not be performed due to the relatively small sample size and limited number of outcome events in several subgroups, which could have resulted in unstable statistical estimates.

Moreover, the incremental value of combining F-18 FDG PET/CT with Ga-68 PSMA PET/CT in staging high-risk prostate cancer is clear from our findings. Dual-tracer imaging may allow for a more comprehensive assessment of both tumor burden and biological activity, which can be crucial for determining the optimal treatment approach. This aligns with the findings of other studies, 19 21 which demonstrate that combining PSMA and FDG imaging enhances detection accuracy and provides complementary information that can guide treatment decisions, particularly for advanced or metastatic prostate cancer.

We believe that the present study contributes clinically relevant information in a specific subset of patients with aggressive prostate cancer. The current study prospectively evaluates biopsy-proven, treatment-naive high-grade prostate adenocarcinoma (ISUP grade groups 4 and 5) using intraindividual dual-tracer PET/CT imaging performed within a short interval. This subgroup represents a biologically heterogeneous and clinically aggressive disease category for which prospective dual-tracer imaging data remain comparatively limited. In contrast to several previous studies that included mixed-risk populations, recurrent disease, metastatic castration-resistant prostate cancer, or retrospective analyses, our study specifically focuses on newly diagnosed high-grade disease.

Additionally, our findings demonstrate the complementary role of F-18 FDG PET/CT in identifying biologically aggressive PSMA-low/negative tumors, particularly in ISUP grade group 5 patients. The identification of discordant PSMA-negative/FDG-positive lesions may have clinical relevance, as these tumors can reflect dedifferentiated disease biology associated with adverse prognosis and potential therapeutic implications.

Although the sample size is relatively modest, prospective intraindividual comparative studies evaluating dual-tracer PET/CT in this high-risk subgroup remain limited, particularly from our geographic region. We believe that the present study contributes additional evidence regarding imaging heterogeneity in aggressive prostate cancer and may provide a basis for larger multicenter prospective investigations.

Our study had few limitations such as small sample size, single institution study, lack of histopathological confirmation for metastatic lesions, no long-term follow-up, and limited patient characteristics as we included only patients with Gleason's score 8 to 10. Certain subgroup analyses including lung, liver, and serosal/peritoneal metastases were underpowered because of the very small number of cases and should therefore be interpreted as descriptive observations rather than definitive comparative analyses. Additionally, multivariate analysis could not be reliably performed due to the relatively small cohort size and risk of statistical overfitting. Therefore, a multicenter prospective study with bigger sample size is required.

## Conclusion

This prospective dual-tracer study demonstrates that Ga-68 PSMA PET/CT remains the dominant imaging modality for detection of primary and metastatic lesions in high-grade prostate adenocarcinoma, particularly for nodal and skeletal disease evaluation. Nevertheless, F-18 FDG PET/CT provided additional clinically relevant information in a subset of biologically aggressive tumors, especially in ISUP grade group 5 and PSMA-low/negative disease phenotypes. The findings support the concept that dual-tracer imaging may improve characterization of tumor heterogeneity and may contribute to individualized imaging-based risk stratification in selected high-risk patients. Further multicenter studies with larger cohorts, histopathological validation, and long-term outcome analysis are warranted to establish the precise clinical role of combined PSMA and FDG PET/CT imaging. While these results are promising, the limitations inherent in our study, including the small sample size, single-center design, and lack of long-term follow-up, suggest the need for further multicenter, prospective studies with larger patient populations and extended follow-up.
